# Evaluation of Centres: the French experience since 2009

**DOI:** 10.1186/1750-1172-9-S1-O4

**Published:** 2014-11-11

**Authors:** S Sarnacki

**Affiliations:** 1 Expert centre on ARM Hopital Necker Enfants Malades, APHP, Paris Descartes University, France

## 

The first French National Rare Disease Plan allowed the establishment of 131 centres of expertise (CEs) from 2004 to 2007. The process of evaluation elaborated with the HAS (Haute Autorité de Santé) was based on a cycle of 5 years: three years after the certification, the CEs were asked for a document of auto-evaluation and at 5 years there was a visit on site to complete the evaluation. As the certification of CEs was done over a period of 4 years, all the CEs were not evaluated at the same time and it resulted in a complex scheme with heterogeneity of the evaluation according to the time point of the process.

The items collected during autoevaluation consisted in general information regarding the composition of the team and of the steering committee, the methods used for the elaboration of the document and quantitative data concerning the activities (clinics, hospitalizations, geographical origin of the patients, proportion of children). Six specific missions of the CEs were also evaluated: expertise, referral, research, epidemiology, healthcare pathway and medicosocial organization. The documented ended with an action plan on which the experts relied for their evaluation during the visit on site at 5 years. This process demonstrated many positive aspects: constructive stimulation of the actors, commitment of institution direction and set up of a virtuous circle based on the fundamental three steps: anticipation, implementation and evaluation. However this process had many caveats: redundancy of some items that led to confusion in the responses, lack of support for the methodology mandatory to evaluate the impact of the CEs actions, heaviness of the organisation of the visit on site and most of all impossibility to retrieve essential information and to analyse them as the documents were elaborated on a word file with free text. The evaluation of the first plan by the high council of public health thus concluded it was necessary to simplify and to optimize the efficiency of the process. In addition, it has to take into account the new ambitions of the 2^nd^ plan.

A working group dedicated to this question collaborated with the HAS and elaborated a simplified document for certification. The process now includes an on-line simplified annual activity report based on quantitative and semi-quantitative data. These reports will be analysed each year by a permanent group – with adjustment of the financial support- together with a final report at 4 years based on the new certification document. Visit on site will be decided only if problems appear during this follow-up or on request of the centre.

